# Automated synthesis and preliminary evaluation of [^18^F]FDPA for cardiac inflammation imaging in rats after myocardial infarction

**DOI:** 10.1038/s41598-020-75705-2

**Published:** 2020-10-29

**Authors:** Tiantian Mou, Jing Tian, Yi Tian, Mingkai Yun, Junqi Li, Wei Dong, Xia Lu, Ziwei Zhu, Hongzhi Mi, Xiaoli Zhang, Xiang Li

**Affiliations:** 1 Department of Nuclear Medicine, Beijing Anzhen Hospital, Capital Medical University, No. 2 Anzhen Road, Chaoyang District, Beijing, 100029 China; 2 Division of Nuclear Medicine, Department of Biomedical Imaging and Image-Guided Therapy, Medical University of Vienna, Vienna, Austria

**Keywords:** Cardiology, Molecular medicine

## Abstract

A translocator protein 18 kDa targeted radiotracer, *N,N*-diethyl-2-(2-(4-[^18^F]fluorophenyl)-5,7-dimethylpyrazolo[1,5-a] pyrimidin-3-yl) acetamide ([^18^F]FDPA), was automated synthetized and evaluated for cardiac inflammation imaging. Various reaction conditions for an automated synthesis were systematically optimized. MicroPET/CT imaging were performed on normal rats and rats with myocardial infarction (MI). Normalized SUV ratios of [^18^F]FDPA to [^13^N]NH_3_ (NSRs) in different regions were calculated to normalize the uptake of [^18^F]FDPA to perfusion. The amount of TBAOMs and the volume/proportion of water were crucial for synthesis. After optimization, the total synthesis time was 68 min. The non-decay corrected radiochemical yields (RCYs) and molar activities were 19.9 ± 1.7% and 169.7 ± 46.5 GBq/μmol, respectively. In normal rats, [^18^F]FDPA showed a high and stable cardiac uptake and fast clearance from other organs. In MI rats, NSRs in the peri-infarct and infarct regions, which were infiltrated with massive inflammatory cells revealed by pathology, were higher than that in the remote region (1.20 ± 0.01 and 1.08 ± 0.10 vs. 0.89 ± 0.05, respectively). [^18^F]FDPA was automated synthesized with high RCYs and molar activities. It showed a high uptake in inflammation regions and offered a wide time window for cardiac imaging, indicating it could be a potential cardiac inflammation imaging agent.

## Introduction

Translocator protein 18 kDa (TSPO) is mainly localized in the outer mitochondrial membrane. It is associated with various biological processes such as controlling the translocation of cholesterol, regulating mitochondrial membrane potential, mediating immune response, modulating voltage dependent calcium channels and apoptosis^1^. Since it is overexpressed in the activated microglia, the TSPO targeted imaging was focused on evaluating neuroinflammation in the past, including Alzheimer’s disease, Parkinson’s disease and dementia^2–4^.

Mitochondria take up 20–30% of the myocardial intracellular volume^5^, making TSPO an attractive biomarker for the diagnosis and evaluation the treatment effects of cardiac diseases^6–8^. Inflammation plays an important role in the healing process after myocardial ischemia^9^. Some clinical trials of anti-inflammatory drugs have been performed on patients with acute myocardial ischemia^10,11^. TSPO is also involved in cardiac inflammation related to macrophage infiltration^12^. Therefore, it might be used to monitor inflammatory response, facilitating physicians to choose the appropriate patients and right time for intervention. Positron emission tomography (PET) is a noninvasive technology to monitor functional and physiological changes in vivo. Currently, several studies reported that TSPO PET imaging could assess cardiac inflammation, such as myocarditis^13,14^ and inflammation after ischemia^15^, making it a “hot target” for cardiac inflammation imaging.

*N,N*-Diethyl-2-(2-(4-[^18^F]fluorophenyl)-5,7-dimethylpyrazolo[1,5-a]pyrimidin-3-yl)acetamide ([^18^F]FDPA) was reported as a TSPO ligand with an excellent binding affinity (*K*_i_ = 2 nM) and a good metabolic stability^16,17^. But the difficulty in fluorine-18 labeling limited its further application. The radiofluorination reaction via [^18^F]fluoride reported in 2015 resulted in low radiochemical conversions (RCCs, < 3%)^17^. Olof Solin, et al. started with carrier-added [^18^F]F_2_ to prepare [^18^F]FDPA. The decay corrected radiochemical yields (RCYs) were increased to 15 ± 3%, but the molar activities were still not satisfied (7.8 ± 0.5 GBq/μmol)^18^. In 2017, Lu Wang, et al. used the adamantyl auxiliary-based precursor 2-(2-(4-(((1*r*,3*r*,5*r*,7*r*)-4′,6′-dioxospiro[adamantane-2,2′-[1,3]dioxan]-5′-ylidene)-λ^3^-iodaneyl)phenyl)-5,7-dimethylpyrazolo[1,5-*a*]pyrimidin-3-yl)-*N,N*-diethylacetamide (DPA-SPIAD) to manually prepare [^18^F]FDPA, and dramatically improved both decay corrected RCYs (45 ± 8%) and molar activities (96 ± 22 GBq/μmol)^19^, making the further evaluation of [^18^F]FDPA possible.

In this report, we optimized an automated synthesis of [^18^F]FDPA based on the previously reported spirocyclic iodonium ylide method^19^, and were first to evaluate [^18^F]FDPA for myocardial inflammation imaging.

## Materials and methods

### Materials

The precursor DPA-SPIAD and the standard compound [^19^F]FDPA were purchased from Shenzhen PET-BiO Technology Co., Ltd. (China). Tetrabutylammonium methanesulfonate (TBAOMs), anhydrous CH_3_CN, and 1-(2-Chlorophenyl)-*N*-methyl-*N*-(1-methylpropyl)-3-isoquinolinecarboxamide (PK11195) were purchased from Sigma-Aldrich (Germany). O-18 enriched water (purity ≥ 98%) was purchased from Jiangsu Huayi Technology Co., Ltd (China). Other chemicals and reagents were purchased from commercial supplies, without further purification.

### Radionuclide production

[^18^F]fluoride was produced via the ^18^O (p, n) ^18^F nuclear reaction by a 11 MeV cyclotron (Eclipse HP, Siemens, USA). A tantalum target containing 2.6 mL O-18 enriched water was irradiated with a proton beam (Target current: 50 μA). At the end of bombardment, [^18^F]fluoride was delivered into a V-vial in CFN-MPS200 Module (Sumitomo Heavy Industries, Ltd., Tokyo, Japan), and then trapped on a QMA exchange solid phase extraction (SPE) cartridge (Waters Corporation, USA), which was pre-activated by 1 mL of 7.4% NaHCO_3_ (aq.) and 10 mL of sterile water.

### Elution efficiency tests

To improve the elution efficiency of [^18^F]fluoride, the stock solution containing different amounts of TBAOMs, water and CH_3_CN was tested manually (Table 1). For each test, 333–555 MBq [^18^F]fluoride was trapped on a QMA cartridge, and measured with a dose calibrator (CRC-25R, Capintec. Inc., USA). Then [^18^F]fluoride was eluted to a vial by different kinds of stock solution, respectively. The radioactivity of [^18^F]fluoride solution was measured subsequently. Elution efficiency = (the radioactivity of [^18^F]fluoride solution / the radioactivity on QMA cartridge) × 100%.

**Table 1:** The elution efficiency of [^18^F]FDPA from the QMA cartridge using different kinds of stock solution (n = 3).

No.	Amount of TBAOMs (mg)	H_2_O (mL)	CH_3_CN (mL)	Elution efficiency (%)
1	12	0.3	0.7	37.6 ± 0.04
2	12	0.3	0.3	79.5 ± 0.03
3	12	0.4	0.6	72.6 ± 0.05
4	12	0.4	0.4	82.3 ± 0.04
5	12	0.4	0.2	85.3 ± 0.01
6	15	0.4	0.4	89.8 ± 0.01
7	20	0.3	0.3	91.9 ± 0.02
8	20	0.4	0.4	98.2 ± 0.01

### Labeling tests

**Figure 1:**
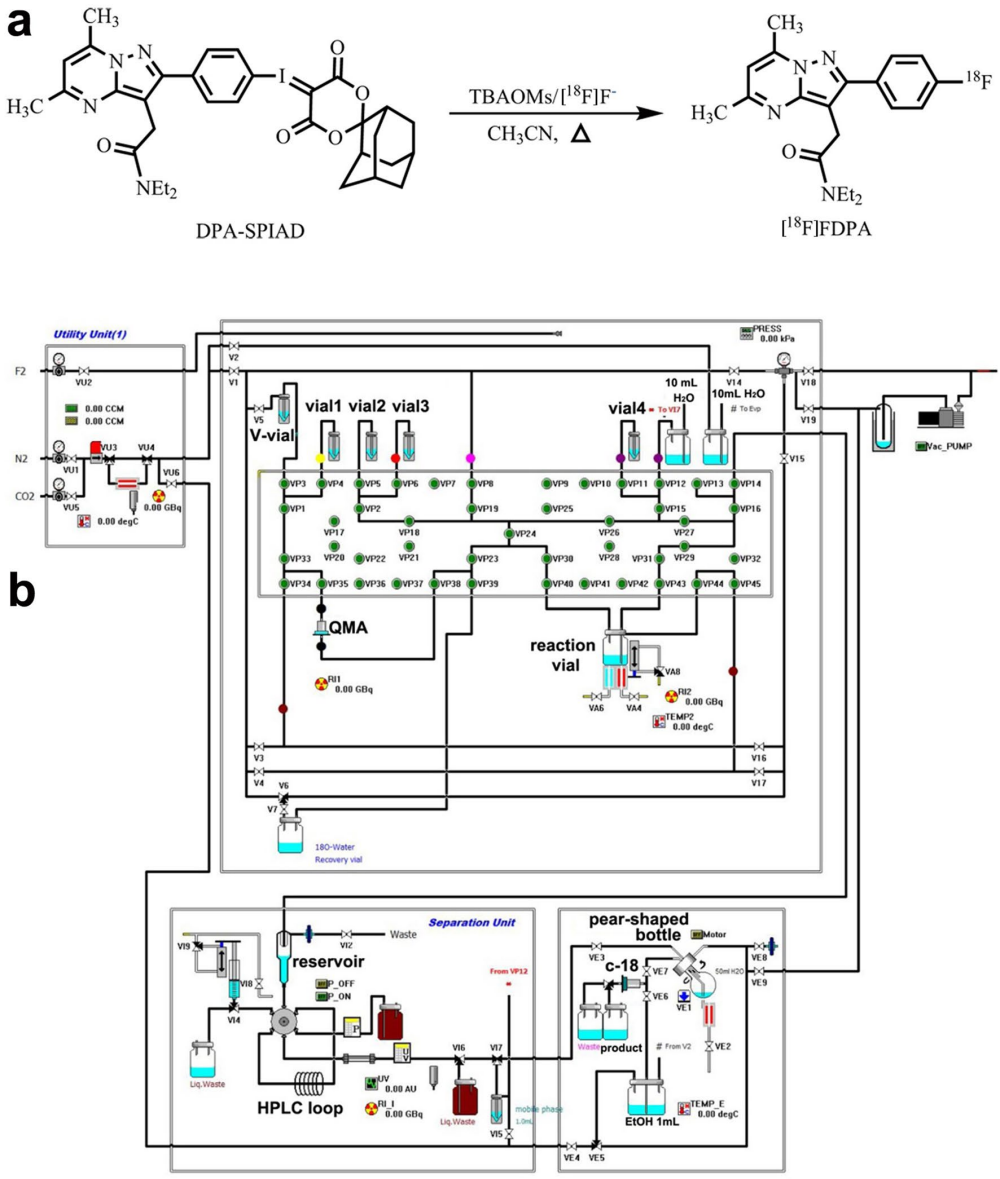
The synthesis route of [^18^F]FDPA. (**a**) The synthesis route of [^18^F]FDPA. (**b**) The diagram of the synthesis cassette of CFN-MPS200 module for [^18^F]FDPA.

The labeling method was shown in Fig. 1. To optimize the automated synthesis process of [^18^F]FDPA, different reaction temperature and reaction time were tested using CFN-MPS200 module. Briefly, 3.7–14.8 GBq [^18^F]fluoride on a QMA cartridge was eluted to the reaction vial with TBAOMs solution (20 mg TBAOMs in 0.4 mL H_2_O and 0.4 mL CH_3_CN), and dried under N_2_ flow at 100 °C. Anhydrous CH_3_CN (0.5 mL) was added, followed by a 4 min evaporation. DPA-SPIAD (2 mg in 1.2 mL CH_3_CN) was added into the reaction vial and reaction was evaluated at different temperatures and maintained for different times (Table 2). After each reaction trial was completed, the reaction mixture was analyzed by HPLC (1260II, Agilent Technologies, USA) to calculate the RCC. The analytical HPLC column (SB-C18, 5 μm, 4.6 × 250 mm, Agilent Technologies, USA) was eluted with 50% 0.1 M ammonium acetate (NH_4_Ac, solvent A) and 50% CH_3_CN (solvent B) at a flow rate of 1 mL/min. The radio-peak for [^18^F]FDPA was confirmed with the corresponding retention time (*t*_R_) determined from the standard compound [^19^F]FDPA. RCC = (the area of radio-peak of [^18^F]FDPA/the summed areas of all radio-peaks) × 100%.

**Table 2:** The RCC of [^18^F]FDPA in different reaction conditions.

#	Reaction temperature (°C)	Reaction time (min)	RCC (%)
1	90	15	4.2
2	100	10	62.7
3	100	15	74.6
4	100	20	50.8
5	120	10	57.4
6	120	15	64.2
7	120	20	40.4

### Adsorption rate of sterile filters

Three kinds of sterile filters, Mtllex-GS (Merck Millipore, Germany), Minisart (Sartorius Stedim Biotech, Germany) and Cathivex-GV (Merck Millipore, Germany) were tested manually. 740–1110 MBq of [^18^F]FDPA in 10% ethanol solution was passed through different filters and collected with a vial, respectively. The radioactivity of filters and [^18^F]FDPA solution in vials was measured soon afterwards. The adsorption rate = (the radioactivity of the filter/the summed radioactivities of the filter and [^18^F]FDPA solution) × 100%.

### Final automated synthesis process

The optimal synthesis process was described as follows (Fig. 1). At the beginning of automated synthesis, [^18^F]fluoride (33.3–40.7 GBq) was trapped on a QMA cartridge, and eluted to the reaction vial with TBAOMs solution (20 mg TBAOMs in 0.4 mL H_2_O and 0.4 mL CH_3_CN) from Vial 1. The solvent was removed by reduced pressure distillation under N_2_ flow at 100 ºC for 7 min. Anhydrous CH_3_CN (0.5 mL) from Vial 2 was added to the reaction vial and heated at 95 °C under N_2_ flow for 4 min. DPA-SPIAD (2 mg in 1.2 mL CH_3_CN) from Vial 3 was added into the reaction vial. Then, the reaction vial was sealed and heated at 100 °C for 15 min. Subsequently, the reaction mixture was cooled to 30 °C and diluted with 1 mL water from Vial 4. The crude reaction mixture was transferred to the reservoir, and then injected onto the HPLC loop with a 2.5 mL syringe. After the automated injection, the semi-preparative column (YMC-Pack ODS-AM C18 semi-preparative column, 250 × 10 mm, 5 µm, YMC Co., Ltd., Japan) was eluted with 50:50 CH_3_CN/0.1 M NH_4_Ac by volume at a flow rate of 4 mL/min. According to ultraviolet (UV, λ = 254 nm) and radiochemical detectors of the module, the desired product (*t*_R_ = 16.5–17.5 min) was collected into a pear-shaped bottle pre-loaded with 50 mL water. The mixture was trapped on a C18 SPE cartridge (Waters Corporation, USA), and washed with 10 mL water. The product was eluted with 1 mL ethanol to product vial subsequently, followed by 10 mL sterile water. The solution was passed through a Cathivex-GV filter, and collected with a 25 mL sterile vial.

The radiochemical purity (RCP) and molar activity of the final [^18^F]FDPA solution were determined by re-injecting the product onto the analytical HPLC column and analyzed with the HPLC method mentioned above. The radioactive fraction was measured by a radio detector (Flow-Count, Bioscan Inc., USA) for molar activity calculation. The mass of the product was calculated by comparing the area under the UV curve at 254 nm with that of standard reference.

### Animal model

Male Sprague–Dawley rats (300–350 g) were purchased from SPF (Beijing) Biotechnology Co., Ltd. (China). Rats were maintained in a temperature-controlled room (25 °C) with a natural day/night cycle and fed with a standard rodent diet and water.

For myocardial infarction (MI) modeling, four rats were anesthetized with an air flow containing 4.0% isoflurane. A left thoracotomy was performed between the third and fourth ribs of the rat. The left pericardium was opened. The left anterior descending branch (LAD) was permanently ligated 1–2 mm below the left atrial appendage, using a 7-0 polypropylene suture with a small curved needle. Successful coronary occlusion was verified by observing the myocardium turned grey after LAD ligation.

All animal experiments were performed according to the laboratory animal management regulations of Beijing, and approved by the Animal Care Committee of Capital Medical University.

### MicroPET/CT imaging protocol

**Figure 2:**
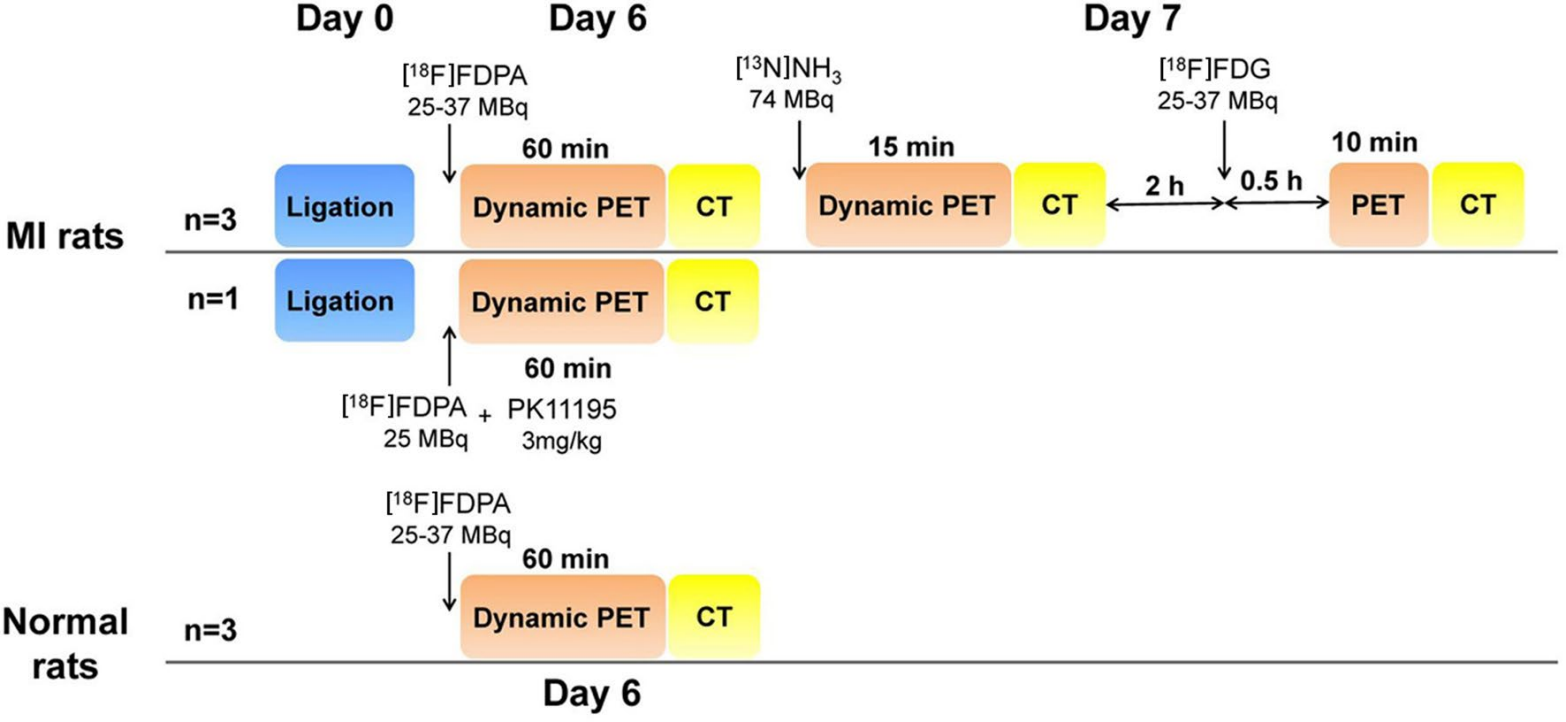
The imaging protocol of microPET/CT with [^18^F]FDPA, [^13^N]NH_3_ and [^18^F]FDG, respectively.

PET/CT imaging studies were performed with a dedicated microPET/CT scanner (Inveon PET/CT, Siemens, Germany). The imaging protocols were shown in Fig. 2.

On the 6th day after surgery, MI rats (350–400 g, *n* = 3) and normal rats (350–400 g, *n* = 3) were performed [^18^F]FDPA imaging study. The rat was placed into a chamber connected to an isoflurane anesthesia unit. Anesthesia was induced using an air flow rate of 2.0 L/min containing 4.0% isoflurane. Then, the animal was immediately placed in a prone position on the scanning bed. The air flow rate was then reduced to 0.8–1.5 L/min with 1–1.5% isoflurane.[^18^F]FDPA (25–37 MBq) was injected via the tail vein. A 60 min dynamic PET scan (6 frames: 6 × 600 s) was started immediately at the beginning of injection, followed by 10 min CT scan using ‘magnification low’ acquisition settings (projection: 180; binning: 4 × 4; transaxial field of view: 53.9 mm; axial scanning length: 134 mm; voltage: 80 kV; current: 500 μA). For the blocking study, a MI rat was injected with a well-known TSPO-selective ligand PK11195 (3 mg/kg) and 25 MBq of [^18^F]FDPA successively. The imaging protocol was same as above.

On the 7th Day after surgery, the MI rats (*n* = 3) were performed [^13^N]NH_3_ imaging study. About 74 MBq [^13^N]NH_3_ was injected via the tail vein. The PET/CT imaging protocol was similar with that of [^18^F]FDPA, except the PET scan lasted for 15 min (2 frames: 1 × 300 s, 1 × 600 s).

Two hours after [^13^N]NH_3_ imaging, the MI rats (*n* = 3) were anesthetized again for [^18^F]FDG imaging. [^18^F]FDG (25–37 MBq) was injected via the tail vein. The PET/CT imaging protocol was similar as that of [^18^F]FDPA, except the PET scan began at 30 min post-injection (p.i.) and lasted for 10 min (1 frame: 1 × 600 s).

### Image reconstruction

The list-mode data of [^18^F]FDPA in normal rats were re-histogrammed to 30 frames of 6 × 20 s, 18 × 60 s, 4 × 300 s, 2 × 600 s for drawing time-activity curves (TACs).

Reconstruction of PET data was performed using a OSEM3D/SP-MAP algorithm (OESM3D: 2 iterations; SP-MAP: 18 iterations; Matrix size: 128 × 128; Image zoom: 1). CT data were reconstructed using a Feldkamp algorithm (Inveon Research Workplace, Siemens, Germany). PET and CT data were co-registered automatically. A 3D-Guassian filter (1.0 mm FWHM) was applied to smooth noise. The reconstructed images were visualized as coronal, sagittal and transversal slices. Regions of organs were carefully drawn according to the CT images. To draw the different regions of the heart, the data of [^18^F]FDPA, [^13^N]NH_3_ and [^18^F]FDG were co-registered by Inveon Research Workplace manually. The infarct region was drawn in the region exhibiting severe defect in both of [^13^N]NH_3_ and [^18^F]FDG images. The peri-infarct region was drawn in the border of infarcted anterior wall where still revealed [^18^F]FDG activity. Mean standardized uptake values (SUVs) were calculated by the software automatically. To normalize the uptake of [^18^F]FDPA to perfusion, the normalized SUV ratio of [^18^F]FDPA to [^13^N]NH_3_ (NSR) was calculated according to the reported procedure with some modification^20^. NSR = (the mean SUV of [^18^F]FDPA in region of interest/the max SUV of [^18^F]FDPA in the heart)/(the mean SUV of [^13^N]NH_3_ in region of interest/the max SUV of [^13^N]NH_3_ in the heart).

### Histology

When imaging studies on the 7th Day after surgery were completed, the rats were sacrificed. The hearts were harvested, and fixed with formalin solution. Each sample was embedded in paraffin, cut into serial sections and mounted on glass slides. Sections were stained with hematoxylin and eosin (H&E)^13^. Tissue sections were examined with a light microscope (Leica DM 3000, Leica, Germany) and processed using Leica Application Suite V4.2 microscope software platform.

### Statistical analysis

IBM SPSS 19.0 was used for statistical analysis. The data was expressed as mean ± standard deviation (SD). SUVs in the heart at different time points were compared using one-way analysis of variance (ANOVA). NSRs in the peri-infarct, infarct and remote regions at 35 min p.i. were analyzed using Kruskal–Wallis H test. A *P* value < 0.05 was considered significant.

## Results

### Optimization of final automated radiosynthesis

As shown in Table 1, when the proportion of water decreased, the elution efficiency decreased remarkably (No.1 vs. No.2, No.3 vs. No.5). Similarly, the stock solution with more volume of water resulted in higher elution efficiency (No.2 vs. No.4, No.7 vs. No.8). On the other hand, more amounts of TBAOMs could improve the elution efficiency as well (No.4 vs. No.6 & No.8). According to Table 1, the best formulation of stock solution was 20 mg TBAOMs in 0.4 mL H_2_O and 0.4 mL CH_3_CN.

Both reaction temperature and time affected RCC (Table 2). The best condition for labeling was heating at 100 °C for 15 min. When the temperature increased to 120 °C, or the reaction time extended to 20 min, RCC reduced dramatically.

[^18^F]FDPA could be adsorbed by some sterile filters, leading to low RCYs. In this study, the adsorption rates of Mtllex-GS, Minisart and Cathivex-GV were 96.3 ± 0.02%, 25.4 ± 0.01% and 3.4 ± 0.01%, respectively (n = 3). Cathivex-GV sterile filter was suitable for [^18^F]FDPA.

### Optimal automated synthesis process

The optimal synthesis process was described above. Starting from [^18^F]fluoride trapped on a QMA cartridge, the total synthesis time was 68 min, including HPLC purification and formulation. The RCYs were 19.9 ± 1.7% (*n* = 3) without correction. The radio-HPLC retention time of [^18^F]FDPA (*t*_R_ = 13.3 min) was consistent with the corresponding nonradioactive reference (*t*_R_ = 12.7 min) (Supplementary Fig. 1). The RCPs ≥ 99%. The molar activities were 169.7 ± 46.5 GBq/μmol (4.6 ± 1.2 Ci/μmol) at the end of synthesis.

### MicroPET/CT imaging

**Figure 3:**
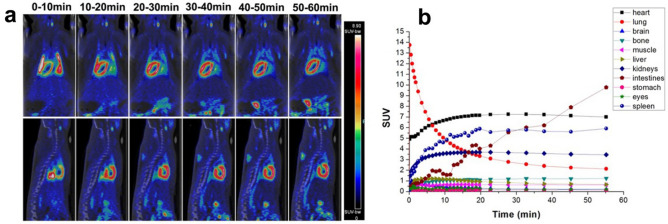
MicroPET/CT imaging studies of [^18^F]FDPA in normal rats. (**a**) Coronal and sagittal PET/CT images from a normal rat at different timepoints p.i. of [^18^F]FDPA. (**b**) Time-activity curves of [^18^F]FDPA obtained from organs of normal rats at 0–60 min p.i., n = 3.

In normal rats, TACs indicated that [^18^F]FDPA mainly accumulated in the heart, lungs, spleen, kidneys and intestines, in which TSPO were highly expressed (Fig. 3). The initial uptake in lungs were too high that the observation of the heart was interfered. Fortunately, the clearance of [^18^F]FDPA from lungs was fast. At 20–55 min p.i., the heart showed stable and prominent uptake of [^18^F]FDPA. The SUVs in the heart were 7.15 ± 0.66 at 20 min and 6.99 ± 0.49 at 55 min p.i. respectively, higher than other organs nearby (Fig. 3). Other tissues in which TSPO were barely expressed, such as muscle (SUVs were 0.63 ± 0.11 at 20 min and 0.62 ± 0.01 at 55 min p.i., respectively), exhibited low uptake of [^18^F]FDPA.

**Figure 4:**
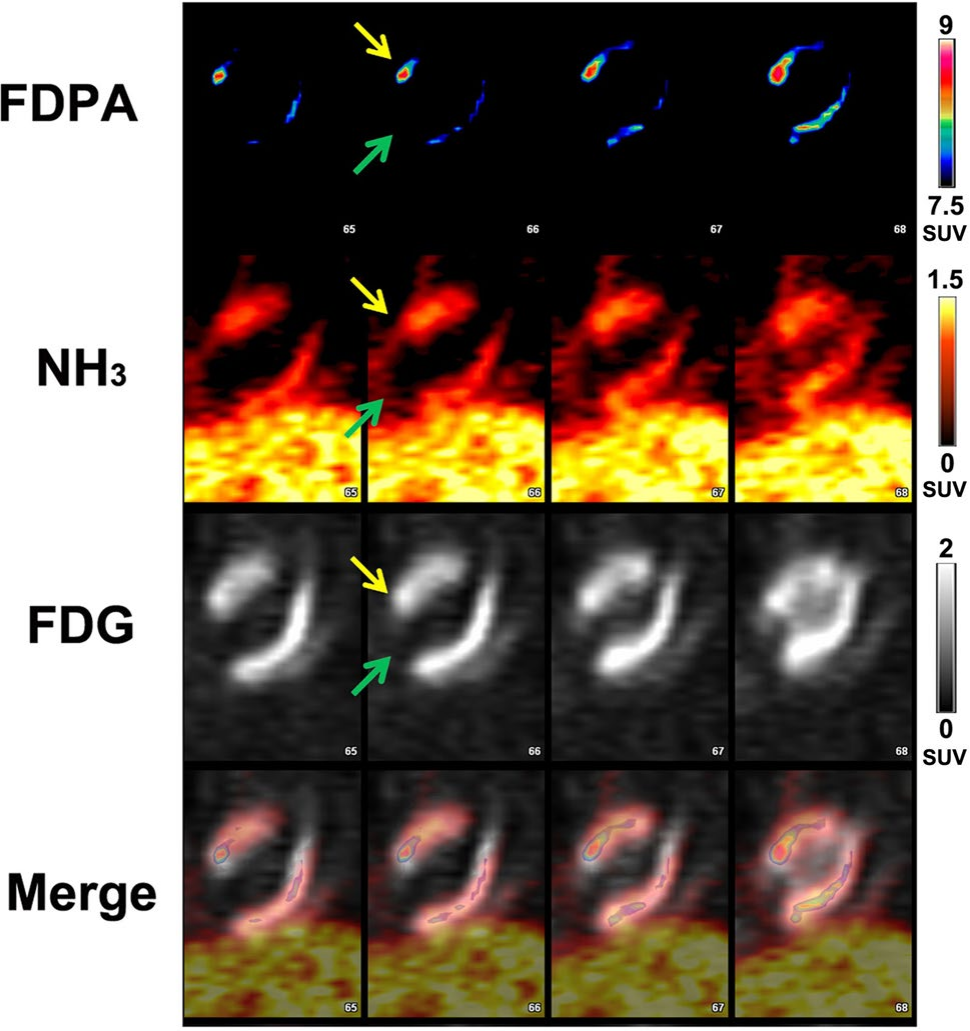
Coronal PET images of a MI rat using [^18^F]FDPA, [^18^F]FDG and [^13^N]NH_3_. The pre-infarct region in the anterior wall (yellow arrows) exhibited a localized elevated [^18^F]FDPA activity, a high [^18^F]FDG activity and a reduced [^13^N]NH_3_ activity. The infarct region in apex (green arrows) showed severe reduced radioactivity in all images.

**Figure 5:**
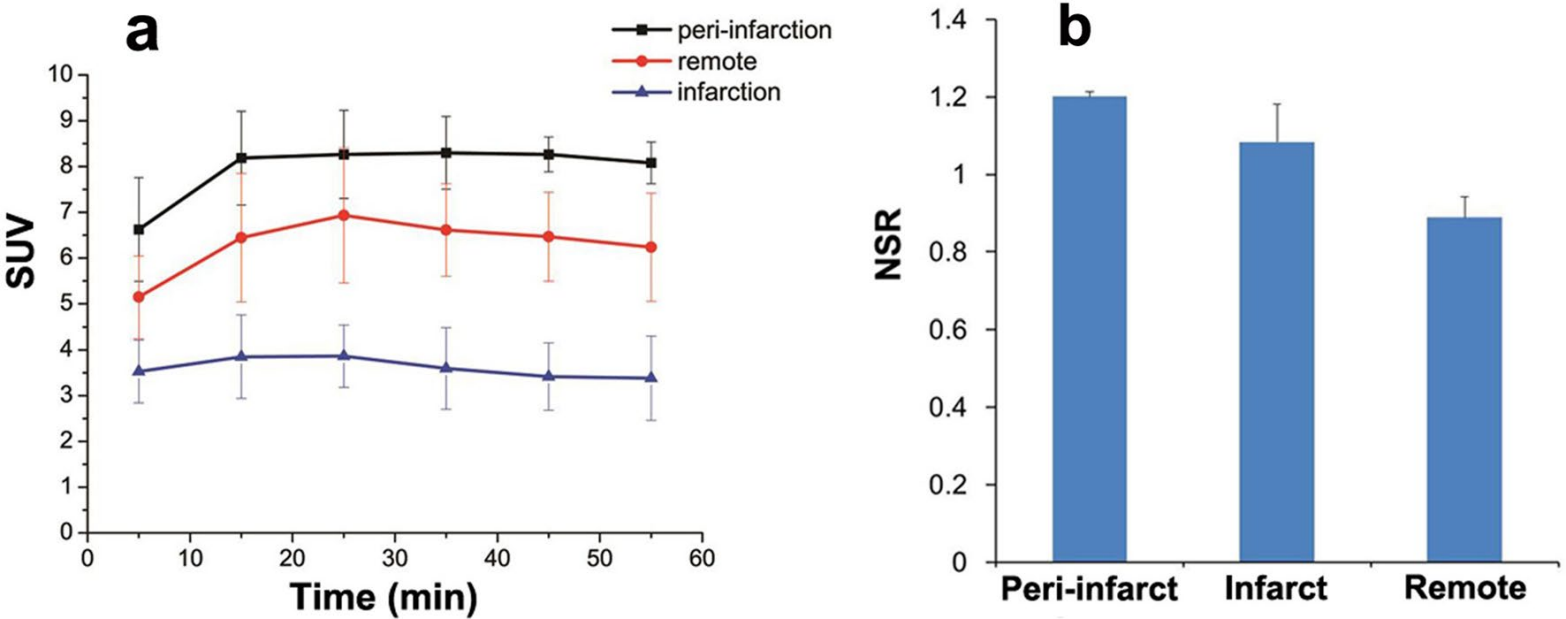
The analysis of radio-uptake in the heart at 0–60 min p.i. (**a**) The SUVs of [^18^F]FDPA in the peri-infarct, infarct and remote regions at 0–60 min p.i.. SUVs were expressed as mean ± SD (n = 3). (**b**) The normalized SUV ratios of [^18^F]FDPA to [^13^N]NH_3_ (NSRs) in the peri-infarct, infarct and remote regions at 35 min p.i. (n = 3).

In the [^13^N]NH_3_ images of MI rats (Fig. 4), a severe perfusion defect with significantly reduced [^13^N]NH_3_ activity was observed in the apex and anterior wall. In [^18^F]FDG images, the extent of reduced [^18^F]FDG uptake was smaller than that in [^13^N]NH_3_ images. As shown in Fig. 5, [^18^F]FDPA was accumulated obviously in peri-infarct region, compared with that in remote region or infarct region at 5–55 min p.i. From 25–55 min p.i., the SUVs in peri-infarct regions were maintained consistently, without significant difference (*F* = 0.064, *P* = 0.977), as well as the SUVs in remote regions (*F* = 0.184, *P* = 0.904) or infarct regions (*F* = 0.220, *P* = 0.880) (Fig. 5). At 35 min p.i., the NSRs in the peri-infarct, infarct and remote regions were 1.20 ± 0.01, 1.08 ± 0.10 and 0.89 ± 0.05, respectively (*P* = 0.027). The NSRs in the peri-infarct, infarct regions were higher than that in remote regions (*P* = 0.022 and 0.539, respectively). In the blocking study, the uptake of [^18^F]FDPA was obviously inhibited by PK11195 (Supplementary Fig. 2), which confirmed the affinity of [^18^F]FDPA to TSPO.

### Histology

**Figure 6:**
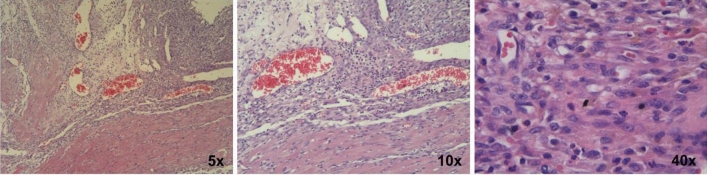
Inflammatory response in the heart of a rat on the 7th day after ligation. Representative H&E histology sections show the infarct and peri-infarct region were infiltrated by inflammatory cells (dark purple stains). The amplification was 5, 10 and 40, respectively.

As shown in Fig. 6, abundant dark purple stains accumulated in the infarct and peri-infarct regions, indicating these regions were infiltrated by inflammatory cells. In contrast, there was few dark purple stains in the remote myocardium.

## Discussion

### Final automated radiosynthesis

[^18^F]FDPA is an analog of a well-known TSPO ligand ^18^F-DPA-714^21,22^. The aryl-^18^F bond of [^18^F]FDPA successfully improved the metabolic stability^18,23^, but also brought a challenge for fluorine-18 labeling. Compared with the manual method^19^, the current study optimized a series of parameters and developed an automated synthesis of [^18^F]FDPA with better RCC (74.6% vs. 63%), higher molar activities (169.7 ± 46.5 vs. 96 ± 22 GBq/μmol), shorter synthesis time (68 min vs 80 min) and comparable RCYs and RCPs^19^. Compared with the automated method reported recently^24^, the process resulted in better RCYs (19.9 ± 1.7% vs. 15.6 ± 4.2%).

In this study, several key points affected the final radiochemical yield from the automated synthesis using this method. Firstly, the amount of TBAOMs and the volume/proportion of water for [^18^F]fluoride elution was crucial to achieving higher RCC and molar activity. Though both the increase of volume or proportion of water can rise elution efficiency, the incremental water may also affect the drying efficiency in the next step. Therefore, we optimized the formulation of stock solution as 20 mg TBAOMs in 0.4 mL CH_3_CN and 0.4 mL H_2_O, instead of the previous method (12 mg TBAOMs in 0.5 mL CH_3_CN and 0.5 mL H_2_O), and resulted in a satisfied elution efficiency. This formulation of stock solution could be a reference for other automated fluorine-18 labeling with TBAOMs. Secondly, the reaction temperature and time also affected the RCC as reported before^19^. In CFN-MPS200 module, the best reaction temperature and time was 100 °C for 15 min. If the reaction time was prolonged or the temperature was increased, RCC decreased accordingly. This may due to the complete evaporation of CH_3_CN under those harsher conditions, which could be observed from the camera of CFN-MPS200 module, leading to no liquid medium available for the reaction. Thirdly, some sterile filters can adversely affect the final radiochemical yield by adsorbing [^18^F]FDPA onto the specific filter membranes preventing the quantitative transfer of radioactivity into the final sterile vial.

### Cardiac inflammation imaging

In the respective PET imaging studies, the absence of radioactivity uptake in the affected MI regions of both [^13^N]NH_3_ and [^18^F]FDG images indicated the MI models were successfully made. Low uptake of [^18^F]FDPA in the liver and the fast clearance from lungs resulted in an improved quality image indicating specific cardiac inflammation when compared to other cardiac inflammation imaging tracers, such as [^125^I]IodoDPA-713^13^, ^18^F-CB251^14^, and ^68^Ga-NOTA-MSA^25^. The stable uptake of [^18^F]FDPA in the peri-infarct, infarct and remote regions offered a wider time window for cardiac imaging. The higher NSRs in the peri-infarct and infarct regions compared with that in the remote regions, combined with the result of H&E staining indicated that [^18^F]FDPA can be accumulated in the inflammation lesions of the heart in MI model.

[^18^F]FDG is used in clinic for cardiac inflammation imaging. However, the high uptake of [^18^F]FDG by cardiomyocytes seriously interfere with inflammation imaging. Dietary strategies or unfractionated heparin administration is required for the suppression of the uptake by viable or normal myocytes^26^. A similar phenomenon is observed in this study. Since TSPO was highly expressed in normal cardiomyocytes^12^, [^18^F]FDPA showed certain uptake in normal myocardium, which might complicate the results of cardiac inflammation imaging. Besides that, in the infarct region, due to the dysfunction of mitochondria in cardiomyocyte^15^, the SUVs of [^18^F]FDPA were not as high as that in remote regions, which affected the visual analysis of inflammation. Therefore, the images of TSPO targeted tracers need an appropriate normalization method for cardiac inflammation imaging. Frank M. Bengel et al. used the polar map of ^99m^Tc-sestamibi as a reference, successfully assessed the elevated signal of a TSPO tracer ^18^F-GE180 in the infarct region of mice at 1 week post MI^15^. In this study, we used the activity of [^13^N]NH_3_ as a reference, calculated the NSRs in the peri-infarct, infarct and remote regions.

TSPO is associated with various cardiac diseases, such as arrhythmia^27^, large vessel vasculitis^28^, cardiac hypertrophy^29^, atherosclerosis^30^ and myocarditis^14^. It is worth mentioning that, TSPO is not only a diagnostic marker, but also a therapeutic target for cardiovascular diseases^12^. TSPO ligands have been studied as therapeutic drugs for cardiovascular diseases, including arrhythmia^27^ and MI^31^. Therefore, the quantitative determination of TSPO level by TSPO targeted tracers may offer more valuable information for individual treatment strategy and curative effects. Besides that, since TSPO targeted tracers are prominent for neuroinflammatory imaging, whole-body TSPO imaging may be used to evaluate the systemic inflammatory response in certain diseases. TSPO targeted tracers are worth further investigations. At the meantime, follow-up studies, especially with suitable methods for analysis, are warranted in the future.

## Conclusion

The synthesis of [^18^F]FDPA was successfully optimized and automated with good RCYs, high molar activities, and short synthesis time. The fast clearance of [^18^F]FDPA from non-target organs and the stable uptake in the heart offered a wider time window for cardiac imaging. Higher NSRs from PET imaging and H&E staining showing the presence of inflammatory cells in the peri-infarct and infarct regions suggest that [^18^F]FDPA could be a potential imaging agent for cardiac inflammation.

## Supplementary information

Supplementary Information.

## Abbreviations

TSPO: Translocator protein 18 kDa
PET: Positron emission tomography
[^18^F]FDPA: *N,N*-Diethyl-2-(2-(4-[^18^F]fluorophenyl)-5,7-dimethylpyrazolo[1,5-a]pyrimidin-3-yl)acetamide
RCC: Radiochemical conversion
RCY: Radiochemical yield
DPA-SPIAD: 2-(2-(4-(((1*r*,3*r*,5*r*,7*r*)-4′,6′-Dioxospiro[adamantane-2,2′-[1,3]dioxan]-5′-ylidene)-λ^3^-iodaneyl)phenyl)-5,7-dimethylpyrazolo[1,5-*a*]pyrimidin-3-yl)-*N,N*-diethylacetamide
TBAOMs: Tetrabutylammonium methanesulfonate
PK11195: 1-(2-Chlorophenyl)-*N*-methyl-*N*-(1-methylpropyl)-3-isoquinolinecarboxamide
SPE: Solid phase extraction
NH_4_Ac: Ammonium acetate
*t*_R_: Retention time
RCP: Radiochemical purity
MI: Myocardial infarction
SUV: Standardized uptake value
NSR: Normalized SUV ratio of [^18^F]FDPA to [^13^N]NH_3_
H&E: Hematoxylin and eosin
SD: Standard deviation
ANOVA: Analysis of variance
