# Supplementary material for: Automated synthesis and preliminary evaluation of [18F]FDPA for cardiac inflammation imaging in rats after myocardial infarction
Source: Sci Rep. 2020 Oct 29;10:18685. doi: 10.1038/s41598-020-75705-2 (PMC7596090; doi:10.1038/s41598-020-75705-2)
Supplement: Supplementary file 1 — Supplementary Information. [file 41598_2020_75705_MOESM1_ESM.doc]

**Supplementary information for**

**Automated Synthesis and Preliminary Evaluation of [^18^F]FDPA for Cardiac Inflammation Imaging in Rats after Myocardial Infarction**

Tiantian Mou^1^#, Jing Tian^1^#, Yi Tian^1^, Mingkai Yun^1^, Junqi Li^1^, Wei Dong^1^, Xia Lu^1^, Ziwei Zhu^1^, Hongzhi Mi^1^, Xiaoli Zhang^1^*, Xiang Li^1,2^

1. Department of Nuclear Medicine, Beijing Anzhen Hospital, Capital Medical University, Beijing, China
2. Division of Nuclear Medicine, Department of Biomedical Imaging and Image-Guided Therapy, Medical University of Vienna, Vienna, Austria


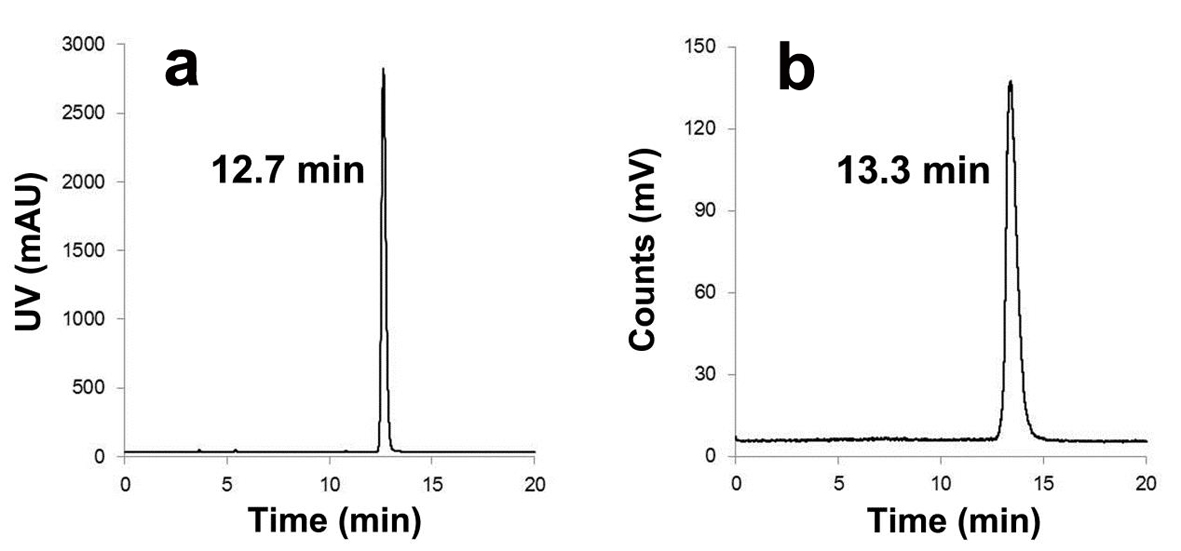


Supplemental Figure 1. HPLC chromatograms of compounds ^19^F-FDPA (a) and ^18^F-FDPA (b) using analytical HPLC method (50% 0.1 M NH_4_OAc and 50% CH_3_CN, at a flow rate of 1 mL/min). Non-radioactive compound ^19^F-FDPA (a) were measured with a UV detector (λ = 254 nm), and ^18^F-FDPA (b) was measured radiometrically.


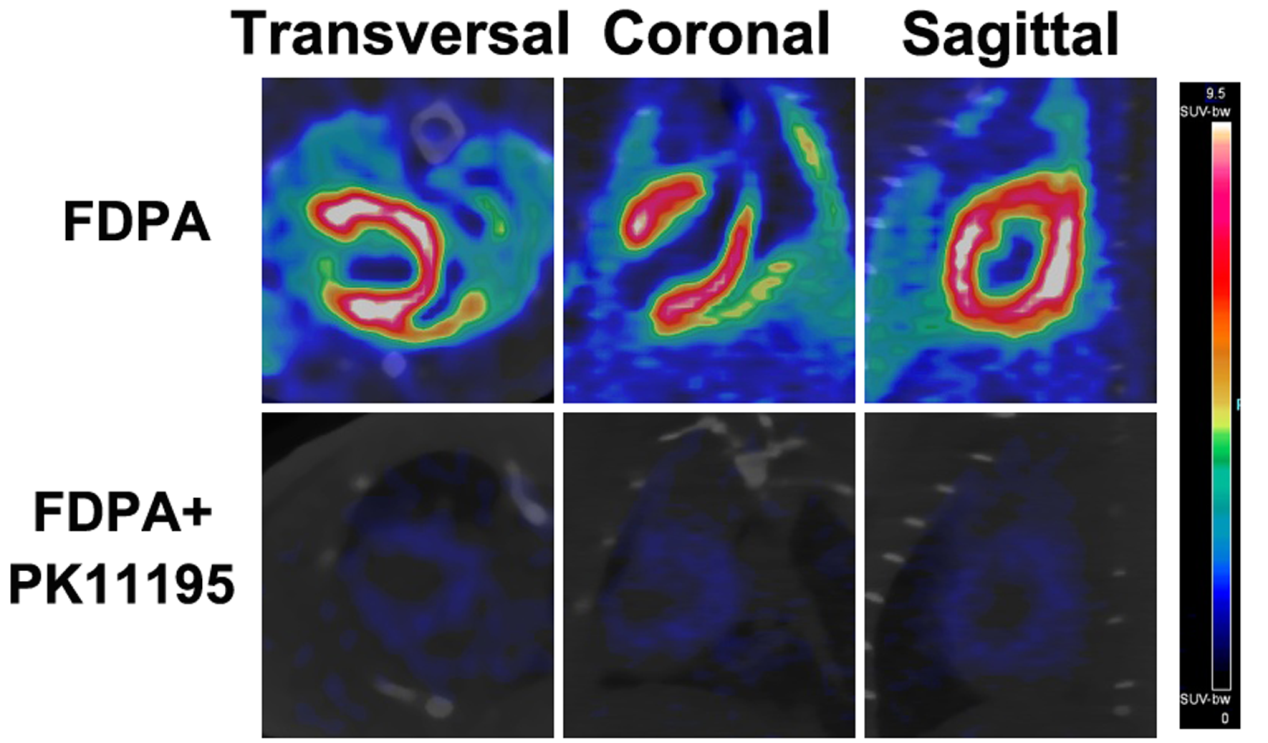


Supplemental Figure 2. Transversal, coronal and sagittal PET images of a MI rat using ^18^F-FDPA and ^18^F-FDPA+PK11195 at 20-30 min p.i..
